# Meat quality characteristics of the Arabian camel (*Camelus dromedarius*) at different ages and post-mortem ageing periods

**DOI:** 10.5713/ajas.19.0589

**Published:** 2019-10-21

**Authors:** Gamaleldin Mustafa Suliman, Abdullah Naser Al-Owaimer, Elsayed Osman Swelum Hussein, Kamaleldin Abuelfatah, Moath Badr Othman

**Affiliations:** 1 Department of Animal Production, College of Food and Agricultural Sciences, King Saud University, P.O Box 2460, 11451 Riyadh, Saudi Arabia; 2 Department of Meat Production, Faculty of Animal Production, University of Khartoum, P.O Box 60, 11451 Khartoum North, Sudan; 3 Department of Agricultural Engineering, College of Food and Agricultural Sciences, King Saud University, P.O Box 2460, 11451 Riyadh, Saudi Arabia

**Keywords:** Arabian Camel, Meat, Age Group, Ageing

## Abstract

**Objective:**

Meat quality characteristics and sensory attributes were evaluated in three age groups (12, 18, and 24 mo) of one-humped camels of the Saudi Arabian Najdi breed.

**Methods:**

Thirty-six male camels (12 for each age group) were used. The *Longissimus dorsi* muscle from each carcass was divided into three parts and subjected to three ageing periods (1, 5, or 10 d) and evaluated for shear force, myofibril fragmentation index (MFI), expressed juice, cooking loss, and sensory attributes.

**Results:**

Age had a significant effect on shear force, MFI, expressed juice quantity, and organoleptic properties. Camels slaughtered at 12 mo exhibited lower shear force and MFI, and higher expressed juice quantity, tenderness, juiciness, and overall acceptability than those slaughtered at 24 mo. Ageing had a significant influence on shear force, MFI, expressed juice quantity, but not on cooking loss. Camel meat aged for 10 d exhibited significantly lower shear force values and expressed juice quantity, and significantly higher MFI compared to that aged for 1 d. However, ageing did not significantly affect sensory attributes, except for tenderness, in camels slaughtered at 18 mo.

**Conclusion:**

Both instrumental and sensory evaluations showed that young camel meat has desirable quality characteristics, with superior tenderness and juiciness.

## INTRODUCTION

The demand for meat protein sources is increasing with increasing population [1]. The camel is a good source of meat, especially in harsh arid and semi-arid areas where climate adversely affects the production efficiency of other animals. The desert camel exhibits great tolerance to high temperatures and water and feed scarcity due to their unique anatomy, physiology, and feeding habits, which have been acquired from a lengthy evolutionary process [2].

The camel meat is rich in animal protein and is an important meat source in many African and Asian countries. In some regions, particularly Arabian countries, camel meat is preferred over meat from other animals, especially in the traditional dishes, due their presumed medicinal benefits [3]. Camel meat is considered healthier than that of other animals due to its lower fat and cholesterol content, as well as for being a good source of minerals, vitamins, bioactive compounds, and essential fatty acids such as n-3 fatty acids [4,5]. There is a common opinion that camel meat is harder, coarser, and more watery than meats from other animals [5]; however, this may be largely due to the fact that camel meat is mostly obtained from old animals that have become less effective in their primary roles of transportation, dairy production, or breeding females [6]. This study was performed to assess meat quality characteristics of relatively young Arabian camel (Najdi) slaughtered at 12, 18, or 24 months of age at different post-mortem ageing periods.

## MATERIALS AND METHODS

### Animal care

This research was carried following the guidelines of work on living animals set by The Research Ethics Committee (REC), King Saud University, Saudi Arabia.

### Animals, slaughtering, and muscle sampling

Thirty-six Saudi Arabian one-humped (Najdi) camels from three age groups (12, 18, and 24 mo), grown in the Riyadh area under the same traditional nutritional and management system consisting of barley grains, alfalfa hay, and wheat straws as the main feed ingredients, were used in this study. Animals were starved for 12 h before slaughtering, with water available at all times. The camels were slaughtered in a local municipal abattoir in Riyadh city, Saudi Arabia. The slaughter process was in accordance with the Islamic legislations as described by Al-Owaimer et al [7]. The entire *longissimus dorsi* muscle was removed, divided into three parts, and each part was aged at 2°C for 1, 5, or 10 d. At the end of the appropriate aging period, the muscles were cut into one-inch steaks, vacuum packaged and stored at −20°C for further analyses.

### Meat quality analysis and sensory evaluation

Shear force measurements were performed according to the procedure described by Wheeler et al [8] using the Texture Analyzer (TA-HD-Stable Micro Systems, Godalming, Surrey, UK) fitted with a Warner-Bratzler attachment. The myofibril fragmentation index (MFI) assay was conducted as described by Culler et al [9]. In brief, 4 g of minced muscle was homogenized in a blender with 40 mL cold (2°C) MFI buffer. After numerous washes, the absorbance of the resultant 0.5 mg/mL solution was measured at 540 nm. The MFI of each sample was calculated by multiplying the absorbance at 540 nm by 200. The expressed juice (EJ) quantity was measured using a filter paper technique and calculated as the total wetted area less the meat area (cm^2^) relative to the weight of the sample (g) [10]. Cooking loss was measured according to the procedure described by Abuelfatah et al [11].

For sensory evaluation, the category scaling method was used to categorize meat samples according to tenderness, juiciness flavor, and overall acceptability on an 8-point category scale. The frozen samples were thawed overnight at 4°C, wrapped in an aluminum foil and cooked in an oven at 163°C for 90 min [11]. Thereafter, the cooked samples were cut into small pieces of approximately 2 cm^3^ and assigned a random code number for identification. The samples were then randomly presented warm (71°C internal temperature) on numbered plates for evaluation. Eight semi-trained panelists were asked to ascribe a category to each meat sample (9 samples per day). The mean of all panel assessments was determined to define the sample characteristic. The panelists were requested to avoid food and smoking 2 h prior to meat tasting. Water was available to remove any residual flavor of the previous samples. No visual or oral communication occurred between panelists during the sensory evaluation.

## RESULTS

### Shear force

**Figure 1:**
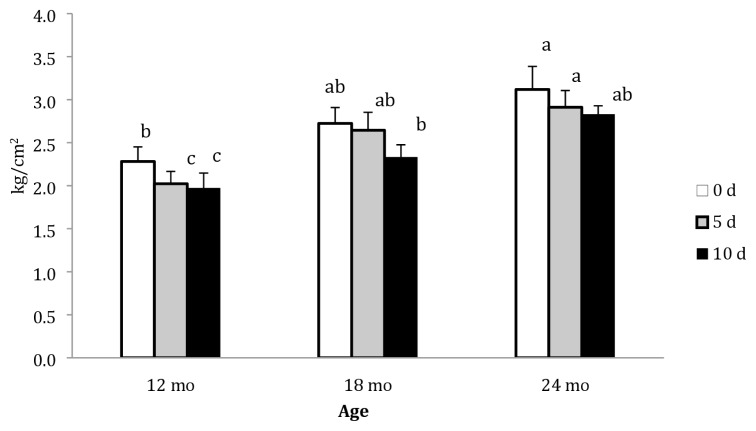
Shear forces (kg/cm^2^) of the *Longissimus dorsi* muscle of Najdi camels at different slaughter ages and ageing periods (d). Bars with different letter indicate statistical significance; error bar = 1 standard error of the mean.

Table 1 shows the effect of age (12, 18, or 24 mo) and post-mortem ageing period (d 0, 5, and 10) on shear force, MFI, EJ quantity, and cooking loss of the *Longissimus dorsi* muscle of Najdi camels. In this study, both age and ageing time factors affected shear force values of camel meat. Shear force increased with increasing age, with a significant difference between 12 and 24 mo age groups, and decreased with increasing post-mortem ageing period. However, the difference between d 5 and d 10 post-mortem ageing period was not significant. Shear force values for different age groups at different ageing periods are illustrated in Figure 1. The highest shear force value (3.12 kg/cm^2^) was recorded in 24 mo group on d 0, whereas the lowest value (2.0 kg/cm^2^) was observed in 12 mo group on d 10. In the 12 mo group, shear force values for d 5 and d 10 ageing periods were significantly higher than that for d 0. In the 18 mo group, shear force values improved with increasing ageing period, but the difference was not statistically significant (p>0.05). Finally, in the 24 mo group, shear force values improved with increasing ageing periods with significant differences between d 0 and d 10.

### Myofibril fragmentation index

**Figure 2:**
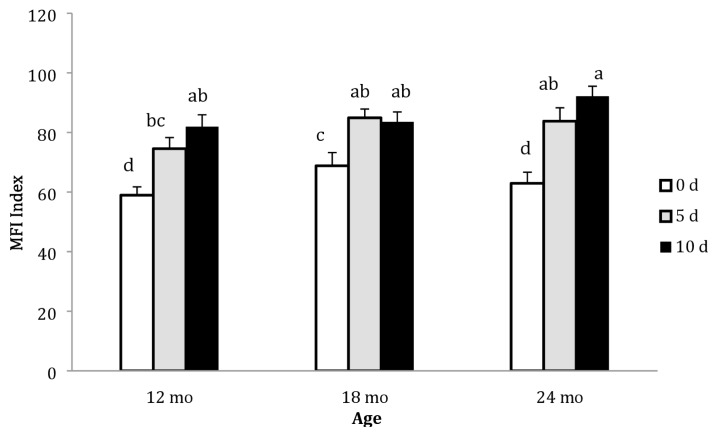
Myofibril fragmentation indices of the *Longissimus dorsi* muscle of Najdi camels at different slaughter ages and ageing periods (d). Bars with different letter indicate statistical significance; error bar = 1 standard error of the mean.

As shown in Table 1, different age groups exhibited different MFI values. The 18 mo group exhibited higher MFI than both 12 and 24 mo groups. Ageing period had a significant impact on the MFI of camel meat. The MFI increased from 64.79 on d 0 to 82.51 and 84.88 on d 5 and d 10, respectively (p<0.05). The MFI values for different age groups at different ageing periods are illustrated in Figure 2. The lowest and highest MFI values were reported for d 0 and d 10, respectively, with no significant difference across different age groups.

### Expressed juice

**Figure 3:**
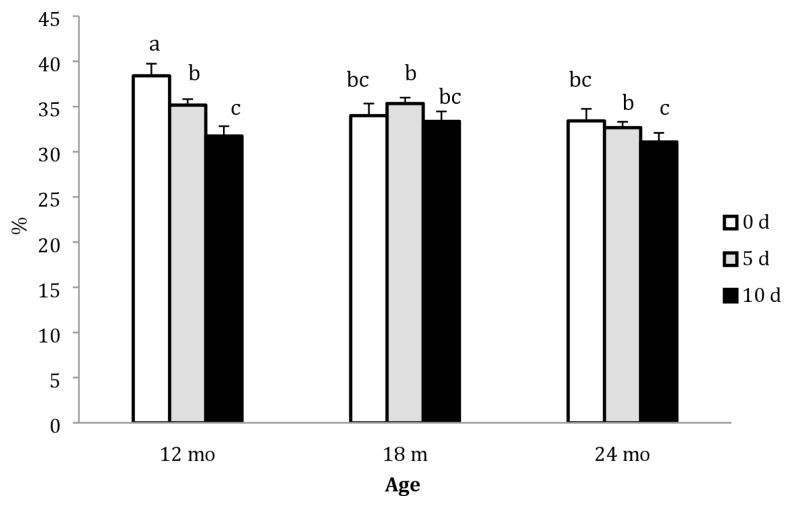
Expressed juice (%) of the *Longissimus dorsi* muscle of Najdi camels at different slaughter ages and ageing periods (d). Bars with different letters indicate statistical significance; error bar = 1 standard error of the mean.

Both age and ageing period significantly affected EJ quantity. Camels in the 12 mo group exhibited higher (p<0.05) EJ quantity than those in the 18 and 24 mo groups. On other hand, EJ quantity significantly decreased with increasing ageing period. The effect of ageing period on expressed juice quantity across different ages is illustrated in Figure 3. The highest EJ quantity (38.4%) was recorded in the 12 mo group on d 0, whereas the lowest value (31.1%) was observed in the 24 mo group on d 10. In the 12 mo group, EJ quantity decreased (p<0.05) with increasing ageing period. However, the difference was not significant in the 18 mo group. In the 24 mo group, a significant decrease in the EJ quantity was observed only on d 10.

### Cooking loss

**Figure 4:**
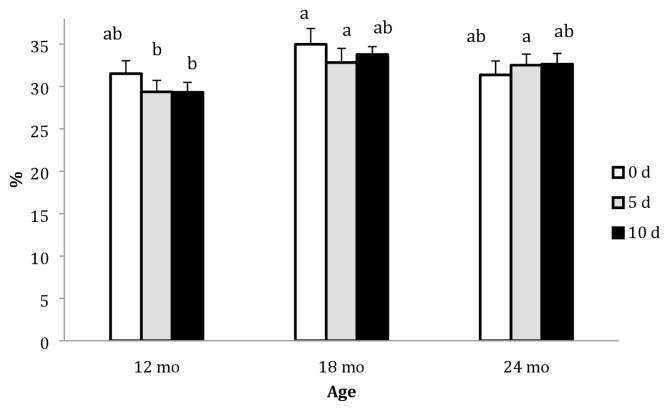
Cooking loss (%) of the *Longissimus dorsi* muscle of Najdi camels at different slaughter ages and ageing periods (d). Bars with different letters indicate statistical significance; error bar = 1 standard error of the mean.

In this study, the cooking loss ranged from 30.16% to 33.25%. Only ageing time produced a significant effect in the 12 mo group, in which the cooking loss for d 5 and d 10 was lower (p<0.05) than that for d 0 (Figure 4).

### Sensory evaluation

The effect of age (12, 18, or 24 mo) and post-mortem ageing period (d 0, 5, or 10) on sensory attributes of cooked camel meat are shown in Table 2. Camels in different age groups showed significant differences regarding meat sensory attributes. Meat samples obtained from 12 mo old camels at different ageing periods scored the highest (p<0.05) points for tenderness, juiciness, overall acceptability (except for d 5), and flavor, but the difference was significant only for d 10.

## DISCUSSION

The objective of this study was to assess quality characteristics of young Arabian camel (Najdi) meat subjected to different post-mortem ageing periods. Meat tenderness is one of the most important quality characteristics that determine consumer acceptability [12,13]. A low shear force value (kg/cm^2^) indicates tender meat and a high shear force value indicates tougher meat. Similar to the results of a previous report by Kadim et al [4], we found that shear force exhibited the most pronounced difference in all meat attributes among different age groups, suggesting that the meat of younger animals is more tender than that of older ones. However, the values of shear force in this study were lower than that reported by Kadim et al [4], Babiker and Yousif [14], Dawood [15], and Jouki and Khazaei [16] in camel meat, in which they ranged from 4.48 to 13 kg/cm^2^. However, the higher values of shear force in these studies can be attributed to the age of the camels, which were slaughtered from 3 to 8 yr. It is generally accepted that younger animals produce meat with lower shear force value than older ones [4]. Moreover, pre- and post-mortem factors and cooking methods affect meat tenderness. Meat ageing can also improve meat tenderness. In this study, we observed that increasing ageing period improves the tenderness of camel meat. A similar result was reported for Iranian camel meat by Jouki and Khazaei [16]. The improvement in meat tenderness by ageing results from the activity of endogenous proteolytic enzymes, which degrade cytoskeletal myofibrillar proteins [17]. The degradation or fragmentation of the cytoskeletal myofibrillar proteins can be monitored by measuring MFI values. There is rational correlation between MFI values and tenderness [18]. The MFI values in this study were in the range reported previously for Omani camel by Kadim et al [4]. The increase in MFI values with increasing ageing period is consistent with similar studies for beef [19]. The findings of the current study support the results that connected between meat tenderness and MFI [20–23].

In this study, the EJ quantity and cooking loss were mea sured to assess the water-holding capacity of camel meat. Water-holding capacity is an important meat quality characteristic because of its influence on the yield and quality of meat [17]. The higher EJ quantities of meat from younger camels (12 mo) than that of 18 or 24 mo old camels were in accordance with the results of previous reports by Kadim et al [4] and Dawood [15]. The effect of ageing period in reducing the EJ quantity in different age groups may be due to degradation of the major cytoskeletal proteins by calpain proteinases [17,24]. In this study, the cooking loss of camel meat across the three age groups at the different ageing periods was in the range of 30% to 33.25%, which is similar to that reported by Babiker and Yousif [14] and Kadim et al [25] in Sudanese and Omani camels, respectively. However, Kadim et al [4] and Kadim et al [26] have previously reported on cooking loss ranging from 26.06% to 22.42%. The variation in cooking loss in different studies can be attributed to many factors, including differences in the cooking method, and ante- and post-mortem handling.

The results of sensory evaluation in this study revealed ac ceptable sensory traits, with scores ranging from 6.48 to 5.12 (sensory panel scales ranged from 8 = extremely desirable to 1 = extremely undesirable). Tenderness refers to the ease of initial penetration by the teeth, followed by breaking into smaller pieces, and finally the amount of residue remaining after mastication. The content and state of the connective tissue, and the structure and state of the myofibrils primarily determine meat tenderness. In the current study, the instrument measurement of camel meat tenderness supports the sensory panel evaluation that younger camels have a more tender meat. Similar to tenderness, juiciness or the ability of cooked meat to release initial and sustained juiciness during chewing was higher in younger camels than that in aged camels. Tenderness and juiciness are closely associated; the more tender the meat is, the faster the juices are secreted and the juicier the meat appears [15].

This study indicates that young camel meat has desirable meat quality characteristics. Both instrumental and sensory evaluations showed that younger camel meat has superior meat quality attributes, especially tenderness and juiciness. Ageing of camel meat has an important influence on MFI and shear force value.

## Figures and Tables

**Table 1:** Effect of slaughter age and post-mortem ageing period on shear force (kg/cm^2^), myofibril fragmentation index, cooking loss (%), and expressed juice (%) quantity of the *Longissimus dorsi* muscle of Najdi camels

Items	Number	Shear force (mean±SD)	MFI (mean±SD)	Expressed juice (mean±SD)	Cooking loss (mean±SD)
Slaughter age (mo)					
12	12	2.17^b^±0.50	71.72^b^±7.32	35.23^a^±2.11	30.16±4.62
18	12	2.48^ab^±0.54	83.14^a^±6.12	33.04^b^±2.55	33.25±5.02
24	12	2.97^a^±0.58	77.31^b^±8. 03	32.54^b^±2.16	32.93±4.65
p-value		0.04	0.01	0.04	0.10
Ageing period (d)					
0	36	2.77^a^±0.70	64.79^b^±5.65	35.57^a^±2.13	33.11±5.71
5	36	2.46^b^±0.64	82.51^a^±5.29	34.20^b^±1.80	31.26±5.07
10	36	2.37^b^±0.57	84.88^a^±6.01	31.59^c^±2.01	31.97±4.25
p-value		0.03	0.01	0.04	0.08

SD, standard deviation; MFI, myofibril fragmentation index.

a–c Means within columns with different superscript letters differ (p<0.05).

**Table 2:** Sensory evaluation of the *Longissimus dorsi* muscle of Najdi camels at different slaughter ages and ageing periods (mean±standard deviation)

Attribute1)	Ageing period (d)	Slaughter age (months)			p-value

		12	18	24	
Tenderness	0	6.48^a^±0.21	5.52^b^^x^±0.25	5.52^b^±0.37	0.03
	5	6.03^a^±0.23	5.53^b^^x^±0.23	5.62^b^±0.41	0.04
	10	6.05^a^±0.41	6.33^a^^y^±0.14	5.43^b^±0.20	0.02
	p-value	0.08	0.03	0.07	-
Juiciness	0	6.53^a^±0.19	5.98^b^±0.25	5.83^b^±0.33	0.04
	5	6.30^a^±0.20	5.52^b^±0.26	5.65^b^±0.32	0.04
	10	6.03^a^±0.36	6.25^a^±0.23	5.12^b^±0.27	0.03
	p-value	0.08	0.06	0.08	-
Flavor	0	5.92±0.24	5.67±0.32	5.82±0.25	0.08
	5	5.98±0.25	5.89±0.20	5.35±0.38	0.10
	10	5.83^a^±0.28	5.72^ab^±0.26	5.15^b^±0.13	0.04
	p-value	0.85	0.59	0.34	-
Overall acceptability	0	6.37^a^±0.20	6.14^a^±0.17	5.70^b^±0.46	0.03
	5	5.86±0.28	5.85±0.20	5.63±0.31	0.87
	10	5.88^a^±0.30	5.80^a^±0.19	5.18^b^±0.29	0.04
	p-value	0.09	0.08	0.25	-

1) Sensory panel scales: range from 8 = extremely desirable to 1 = extremely undesirable.

a,b Means within rows with different superscripts differ among camel age (p<0.05).

x,y Means within columns with different superscripts differ among post-mortem ageing periods (p<0.05).
